# A nested variant of urothelial carcinoma of the urethra and urinary bladder: A case report and review of literature

**DOI:** 10.1002/ccr3.7360

**Published:** 2023-05-25

**Authors:** Ali Taghizadeh Kermani, Mahmoud Reza Kalantari, Zohreh Pishevar Feizabad, Soudeh Arastouei

**Affiliations:** ^1^ Cancer Research Center Mashhad University of Medical Sciences Mashhad Iran; ^2^ Department of Pathology, Faculty of medicine Mashhad University of Medical Sciences Mashhad Iran

**Keywords:** bladder neoplasm, chemotherapy, nested variant, urothelial carcinoma

## Abstract

Nested variant of urothelial carcinoma (NV‐UC) is an extremely rare cancer with a nonspecific presentation. It is usually identified at a late stage, which makes the treatment challenging. Herein we report the case of a 52‐year‐old woman with an advanced NV‐UC treated by anterior exenteration after a poor response to neoadjuvant chemotherapy. One year after completion of adjuvant radiotherapy, the patient remains disease‐free.

## Graphical abstract

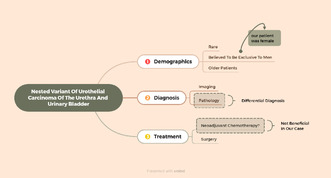

## 1 INTRODUCTION

Nested variant of bladder urothelial carcinoma (UC‐NV) is a relatively rare but aggressive neoplasm, characterized by confluent small nests of mildly atypical neoplastic cells, infiltrating the lamina propria or muscularis propria of the bladder.^1^ Limited cases have been reported so far. This study aims to discuss the clinical, histological, immunohistochemical, and treatment profiles of this rare neoplasm by describing a case of a nested variant of urothelial carcinoma of the urethra in a female patient.

## 2 CASE PRESENTATION

A 52‐year‐old female patient, with a family history of bladder cancer in her older brother, presented to a urologist in November 2021, with a history of dysuria and frequency. She had been treated with a presumptive diagnosis of urinary tract infection twice, empirically. Past medical history revealed she was a non‐smoker with a history of hyperlipidemia and asthma. Her brother was diagnosed with bladder cancer at 60 years of age. On physical examination, she had no organomegaly or lymphadenopathy.

On initial work up, her urine cytology was negative for atypical cells, with a negative urine culture. Abdominal ultrasound demonstrated a 46 × 35 mm solid hypoechoic mass in the urethra and bladder neck. Pelvic magnetic resonance imaging (MRI) revealed a urethral mass with extension to the bladder neck, vagina, and the left levator ani muscle, along with a prominent lymph node in the obturator region, which was suspicious for metastases.

**FIGURE 1:**
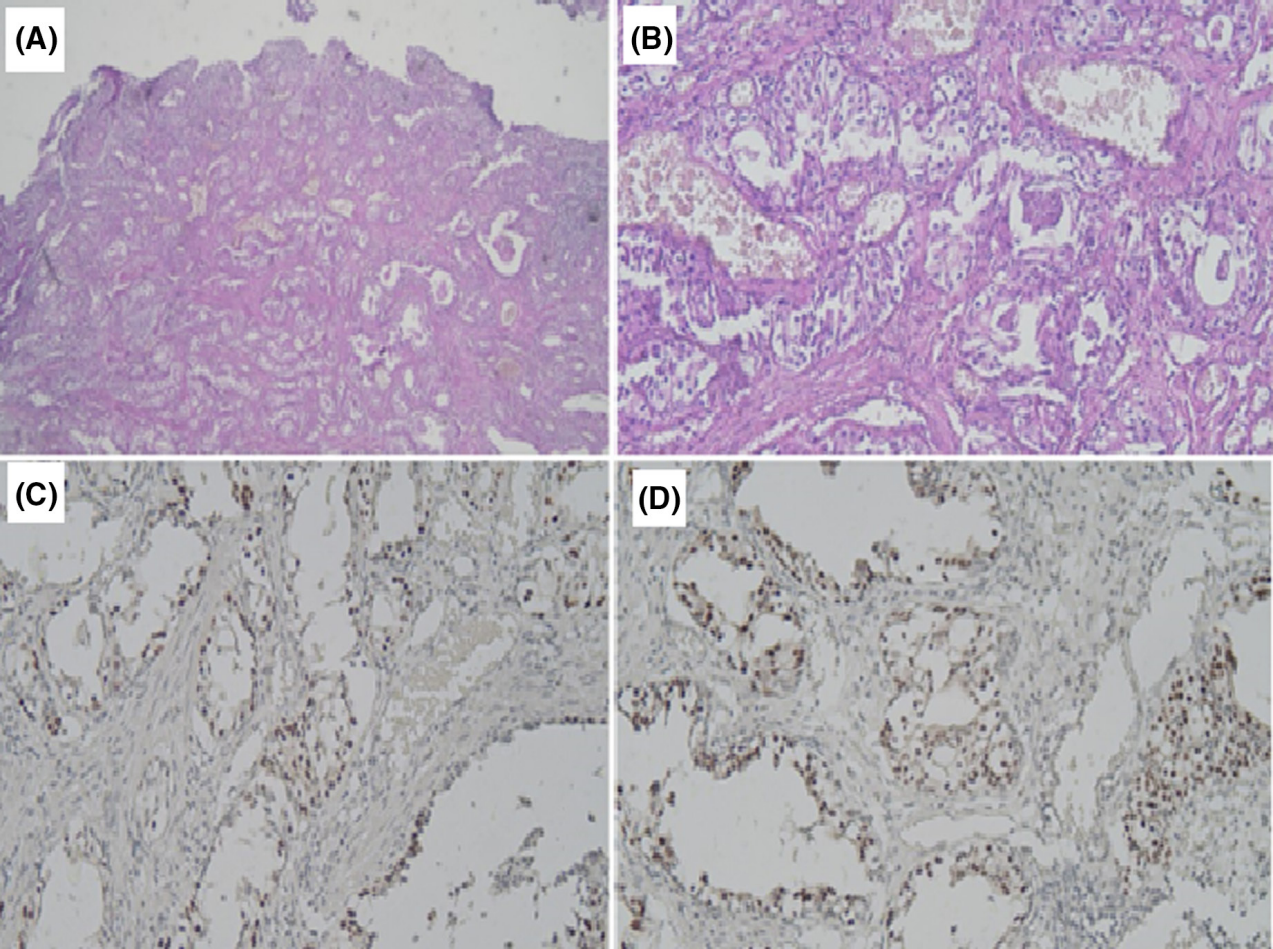
Histologic features of tumor tissue. (A, B) Hematoxylin and eosin staining show epithelial cell nests, some with central lumen and some with microcystic changes, compatible with aggressive nested variant of urothelial carcinoma, with invasion to underlying detrusor muscle. The neoplastic cells reveal low cytonuclear atypia. (C, D) C showcases positive immunoreactivity of tumoral cells for P63. D shows immunoreactivity of tumoral cells for GATA3.

A transvaginal core needle biopsy was done, which came back highly suspicious for infiltrating carcinoma. Transurethral resection of bladder tumor (TURBT) was performed. Histologic examination of the biopsy specimen was compatible with aggressive, invasive nested variant of urothelial carcinoma, which was confirmed by a second pathologist. On immunohistochemical (IHC) examination, tumoral cells were positive for p63 and GATA3 (Figure 1).

**FIGURE 2:**
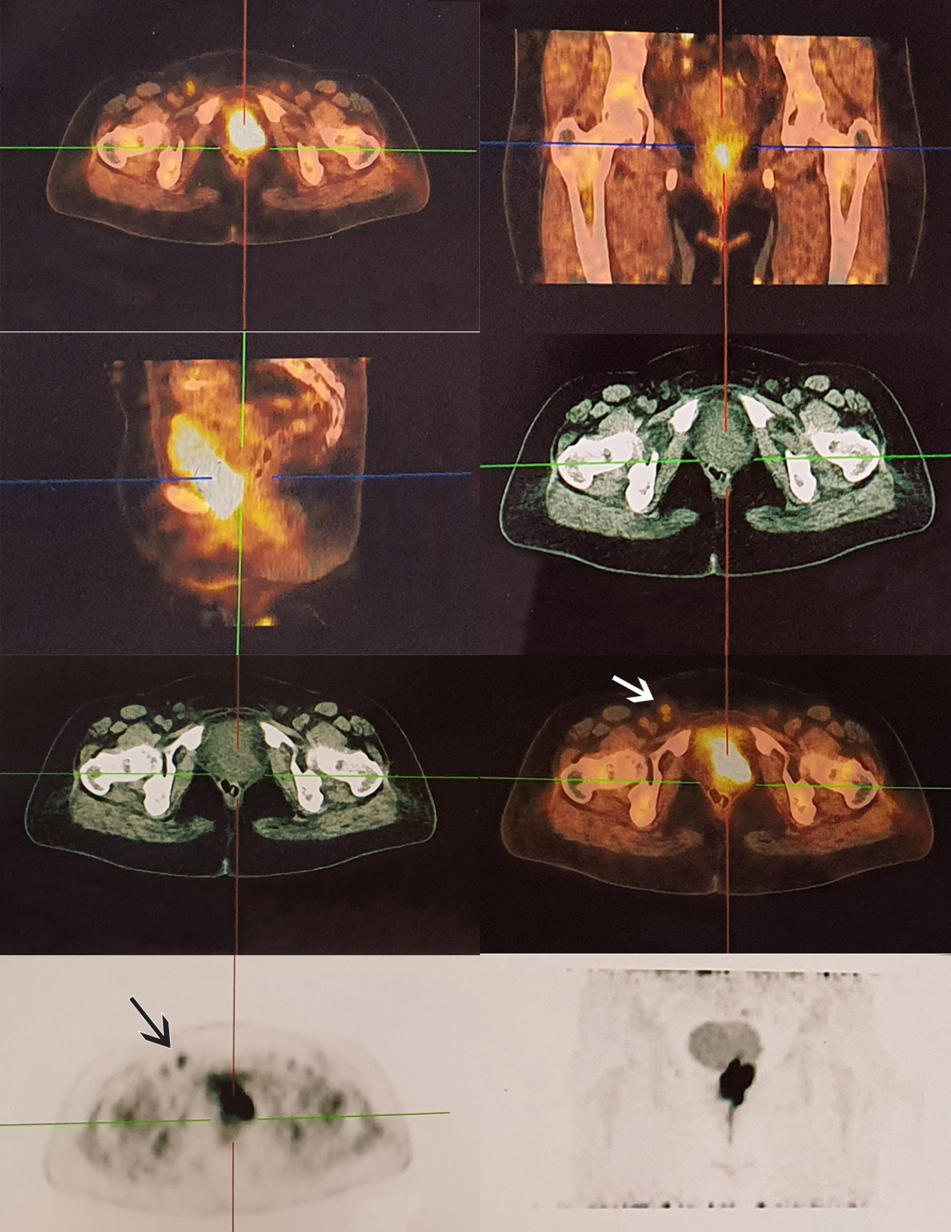
FDG‐PET/CT scan shows a hypermetabolic tumoral mass in the urethra with invasion to the vagina. Few hypermetabolic lymph nodes are noted in bilateral inguinal regions, more prominently on the left side in favor of lymph node metastasis.

Whole body FDG‐PET/CT scan showed a hypermetabolic tumoral mass in the urethra with invasion into the vagina, and a few hypermetabolic lymph nodes in the bilateral inguinal regions, which was suspicious for lymph node metastasis (Figure 2). An ultrasound‐guided needle biopsy of left inguinal adenopathy revealed it to be a reactive lymph node.

After presenting the case in a multidisciplinary care team and discussing the advantages and disadvantages of all available options, she was treated by neoadjuvant chemotherapy (NACT), with 3 cycles of gemcitabine 1 g/m^2^ (on Days 1 and 8) and cisplatin 70 mg/m^2^ (on Days 1 and 2) every 3 weeks. After obtaining a written informed consent, the patient underwent surgery. Intraoperatively, the persistence of a huge urethral mass, with invasion of bladder trigone was detected. As the frozen section revealed involvement of periurethral and pericervical tissue, an anterior exenteration was performed. The procedure included radical cystectomy, along with total hysterectomy, bilateral salpingo‐oopherectomy, and bilateral pelvic lymphadenectomy with institution of an ileal conduit. Postoperatively, the patient was admitted to intensive care unit (ICU) for a day. She had an uneventful course and was discharged from hospital one week later.

On pathological examination, the periurethral tissue and distal urethra were focally involved by the tumor. The patient was further discussed in the multidisciplinary team, and considering the pros and cons of all available adjuvant options, adjuvant radiotherapy was recommended. Therefore, using intensity‐modulated radiation therapy (IMRT) technique, she was treated with a total dose of 5940 cGy at 180 cGy per fraction as adjuvant treatment. The target volumes included the surgical bed, the lower part of the common iliac, external and internal iliac, inguinal, and obturator nodal regions, with localization to surgical bed at 4500 cGy. Organs at risk included: femoral heads, rectum, bowel bag, and ileal conduit. As there are no guidelines specifying the tolerance limit for the ileal conduit, efforts were made for the conduit to receive the minimum possible dose.

Thereafter, the patient was followed with history, physical examination, and imaging every 3 months. In her second follow‐up visit, she complained of rectal bleeding, which was evaluated by clinical examination and colonoscopy, indicating a radiation induced proctitis. Her latest CT scan, 12 months post adjuvant treatment, is free from disease.

## 3 DISCUSSION

Urothelial carcinoma of the bladder is the most common malignancy of the urinary system. Less commonly, urothelial carcinoma can occur in the renal pelvis, ureter, and urethra.^2^ Based on the degree of nuclear anaplasia and architectural abnormalities, this tumor has been divided into low‐grade and high‐grade tumors. Histologic variants of urothelial carcinoma include lymphoepithelioma‐like cell, sarcomatous, plasmacytoid, microcystic, micropapillary, nested, and small cell variants.^2^

The nested variant of urothelial carcinoma (UC‐NV) is characterized by a bland morphology mimicking some benign urinary bladder lesions like von Brunn's nests, but it has a clinical behavior of high‐grade conventional urothelial carcinomas.^3^ There is a tendency that as cellular anaplasia increases, depth of invasion also increases.^3^ Infiltration into the muscularis propria is often associated with higher cytologic atypia.^4^ The neoplastic cells in the lamina propria are dominated by slightly atypical, cuboidal cells with pale cytoplasm, rectangular nuclei, and inconspicuous nucleoli. These cells are usually arranged in small and round nests or tubules. Occasionally, a trabecular pattern is seen.^2^, ^5^ In our case the tumoral cells showcased a low cytonuclear atypia, but the tumor was aggressive.

NV was first described as a benign tumor in 1979,^6^ and it was only in 1989, it was identified as a malignant tumor by Talbert et al.^7^ Few cases of the disease have been reported so far. The average age at the time of diagnosis is 68.7 years (range, 45–97 years); however, our case was younger than average. Contrary to the present case, most cases present in males which perpetuates the misconception that this tumor is almost exclusively seen in men.^5^

Gross hematuria is the most common presenting sign.^5^ In our case, the patient presented with symptoms mimicking a urinary tract infection, which delayed the diagnosis. The periureteral orifice is a major site for TCC‐NVs, with the tumor usually located close to the bladder neck and ureteric ostium.^8^ The overrepresentation of UC‐NV in the trigone could be due to the prevalence of von Brunn's nests in this area, as these tumors primarily arise in von Brunn's nests.^9^

The differential diagnosis for NV‐UC includes the proliferation of Brunn's nests, cystitis cystica, cystitis glandularis, proliferative cystitis, inverted papilloma, adenocarcinoma, nephrogenic metaplasia, and paraganglioma.^7^ Irregular distribution, presence of large numbers of closely packed epithelial aggregates and deep invasion, together with focal, mild to moderate cytologic atypia distinguish these foci from the benign conditions mentioned.^3^ Also, a tendency toward increasing cellular anaplasia in the deeper portions of the lesion may help distinguish the NV‐UC from the benign conditions.^10^ IHC staining with PSA, neuron‐specific enolase, and chromogranin are useful for narrowing down the differential diagnosis. UC‐NV is associated with high Ki‐67 labeling index.^3^, ^5^ The nested component demonstrates an immunophenotype identical to usual UC, with CK7, CK20, p63, and CK903 expression in 93%, 68%, 92%, and 92% of cases, respectively.^4^ UC‐NV can be differentiated from prostate carcinoma by a negative PSA reaction.^5^

It is also important to differentiate the usual UC involving von Brunn's nests from UC‐NV. Von Brunn's nests are most commonly observed in the trigone and consist of clusters of urothelial cells within the lamina propria that have been detached from the epithelium above. They are found in 80–90% of normal bladders.^8^

Tumor prognosis is considered relatively poor, especially with vascular or lymphatic invasion.^3^, ^5^ Long survival is seen with early stage of the disease, while those with distant metastases show dismal survival.^11^ When stagematched, the 10‐year survival and recurrence rate is similar to pure urothelial carcinoma.^12^

Since most patients are diagnosed at advanced stages, they are generally treated with NACT and radical cystectomy.^13^ The effect of NACT, which has demonstrated significant survival benefit in urothelial carcinoma, is unknown in these rare subtypes. Hajiran and colleagues retrospectively reviewed 768 patients with clinical muscle invasive bladder cancer, 173 with variant histology, and reported that similar response rates were seen in all pathologies, and that both pure urothelial carcinoma and variant histology cohort demonstrated a survival benefit with this approach.^14^ In a recent narrative review, the use of NACT is recommended for pathologic downstaging, as a higher pathologic stage on surgery portends a worse survival outcome.^15^ Based on this data, our patient was put on NACT, which was not beneficial in her case.

In conclusion, diagnosis and treatment of NV‐UC are challenging due to its nonspecific clinical finding and low incidence. While it might be difficult to differentiate the tumor from benign conditions initially, an early diagnosis with biopsy especially using immunohistochemical assay, may improves survival rates. Despite the current recommendations regarding benefits of NACT in the management of patients with NV‐UC to reduce the tumor volume, the responses to NACT were not significant in our case. Therefore, considering the paucity of evidence, patients scheduled to receive NACT should be evaluated carefully during the treatments for discontinuation of NACT if there is a poor response to avoid further toxicities. Moreover, novel therapeutic agents are needed to improve the survival of these cases.

## AUTHOR CONTRIBUTIONS

**Ali Taghizadeh Kermani:** Conceptualization; supervision; writing – original draft. **Mahmoud Reza Kalantari:** Resources; validation; visualization; writing – review and editing. **Zohreh Pishevar Feizabad:** Conceptualization; resources; validation; writing – review and editing. **Soudeh Arastouei:** Conceptualization; data curation; supervision; writing – original draft; writing – review and editing.

## FUNDING INFORMATION

The authors received no specific funding for this work.

## CONFLICT OF INTEREST STATEMENT

The authors have no conflict of interest to declare.

## CONSENT

A written informed consent was obtained from the patient for publication of her clinical details and images in accordance with the journal's patient consent policy.

## Data Availability

The data that support the findings of this study are available from the corresponding author upon request.
